# Epidermal growth factor (EGF) and interleukin (IL)-1β synergistically promote ERK1/2-mediated invasive breast ductal cancer cell migration and invasion

**DOI:** 10.1186/1476-4598-11-79

**Published:** 2012-10-21

**Authors:** Liqiang Ma, Fenghua Lan, Zhiyong Zheng, Feilai Xie, Lie Wang, Wei Liu, Junyong Han, Feng Zheng, Yanchuan Xie, Qiaojia Huang

**Affiliations:** 1 Institute for Laboratory Medicine, Fuzhou General Hospital, Second Military Medical University, 156 North Xi-er Huan Road, Fuzhou City, Fujian Province, 350025, China; 2 Department of Pathology, Fuzhou General Hospital, Second Military Medical University, 156 North Xi-er Huan Road, Fuzhou City, Fujian Province, 350025, China; 3 Department of General Surgery, Fuzhou General Hospital, Second Military Medical University, 156 North Xi-er Huan Road, Fuzhou City, Fujian Province, 350025, China; 4 Department of Nephrology, Fuzhou General Hospital, Second Military Medical University, 156 North Xi-er Huan Road, Fuzhou City, Fujian Province, 350025, China

**Keywords:** EGF, IL-1β, ERK1/2, Invasive breast ductal carcinoma, BT-474 cells, Metastasis, MMP-9, AP-1

## Abstract

**Background:**

Patients with invasive breast ductal carcinoma (IBDC) with metastasis have a very poor prognosis. Little is known about the synergistic action of growth and inflammatory factors in IBDC metastases.

**Methods:**

The expression of activated extracellular signal-regulated kinase1/2 (phosphorylated or p-ERK1/2) was analyzed by immunohistochemistry in IBDC tissue samples from 80 cases. BT474 IBDC cell migration and invasion were quantified using the Transwell assay. Matrix metalloproteinase (MMP)-9 expression and activity were analyzed by RT-PCR, Western blotting and zymography. Activator protein (AP)-1 activity was measured with a luciferase reporter gene assay. The Wilcoxon signed-rank test, Chi-square test, the partition of Chi-square test, independent t-test, and Spearman’s method were used for the statistical analysis.

**Results:**

Phosphorylated ERK1/2 was detected in 58/80 (72.5%) IBDC tissues, and was associated with higher TNM stage and lymph node metastasis, but not patient age or tumor size. Individually, epidermal growth factor (EGF), and interleukin (IL)-1β activated ERK1/2, increased cell migration and invasion, MMP-9 expression and activity, AP-1 activation in *vitro* and the expression of p-ERK1/2 was positively correlated with EGF expression levels, as well as IL-1β, MMP-9 and c-fos in IBDC tissue samples. Co-stimulation with EGF and IL-1β synergistically increased ERK1/2 and AP-1 activation, cell migration and invasion, and MMP-9 expression and activity. Inhibition of ERK1/2 using U0126 or siRNA abolished EGF and/or IL-1β-induced cell migration and invasion in a dose-dependent manner.

**Conclusion:**

Activated ERK1/2 was associated with higher TNM stage and lymph node metastasis in IBDC. Both in *vitro* and *in vivo* studies indicated that ERK-1/2 activation may increase the metastatic ability of IBDC cells. Growth and inflammatory factors synergistically induced IBDC cell migration and invasion via ERK1/2 signaling, AP-1 activation and MMP-9 upregulation.

## Background

Breast cancer is one of the most common malignancies in women and represents 22.9% of all female cancers worldwide [1]. Although the 5-year survival rates for breast cancer throughout the world are generally good [1,2], the prognosis for patients with metastases is very poor, especially in metastatic invasive breast ductal carcinoma (IBDC). The processes by which metastasis occurs in breast cancer are still poorly understood; therefore, characterization of the precise molecular mechanisms that regulate metastasis in breast cancer could potentially result in a large reduction in the number of breast cancer deaths and may lead to novel treatments for breast cancer.

The mitogen-activated protein kinase (MAPK) signaling pathways have been widely studied, and contain at least three MAPK superfamilies that regulate diverse cellular activities. Extracellular signal-regulated kinase (ERK) is the most essential MAPK signaling pathway and is involved in cell growth, motility and survival [3,4]. Five ERK homologs have been identified: ERK1, ERK2, ERK5, ERK7 and ERK8 [5,6]. It is well established that ERK1 and ERK2 are two of the most important regulators of cell proliferation, growth, differentiation and migration [7,8], and these processes are closely related to cancer cell progression. MAPK is activated by the upstream MAP2K kinases, which in turn are activated by the MAP3K kinases. To date, several MAP3Ks and MAP2Ks have been identified that regulate the ERK signaling pathway, including the MAP3Ks Raf and Mos, and the MAP2Ks, MAPK/ERK kinase 1 (MEK1) and MEK2. ERK1/2 is a direct target of MEK1 and MEK2 [9].

ERK1/2 activation has been observed in a wide variety of cancers, and is closely associated with the development of human cancer and also with the migration, invasion and metastasis of cancer cells [10]. Therefore, the ERK1/2 signaling pathways are regarded as potential targets for new cancer treatments [11]. ERK1/2 is frequently activated by growth factors, such as epidermal growth factor (EGF), which leads to increased cell growth, differentiation and migration. Although the EGF/Raf/MEK1/2/ERK1/2 pathway has been investigated with respect to cancer cell metastasis, and it is known that the activation of ERK1/2 promotes the growth of breast cancer cells [12], the effect of ERK1/2 signaling activation on the metastasis of invasive breast ductal carcinoma is poorly characterized and remains of interest.

Recently, increasing attention has been paid to the tumor microenvironment, which has been closely associated with carcinogenesis and metastasis [13,14]. Accumulating evidence demonstrates that the cytokines secreted by tumor cells are important components of the tumor microenvironment. Interleukin (IL)-1β can activate ERK1/2 in several different cell types, and can also activate the transcription factor activator protein (AP)-1, which may promote inflammation-associated carcinogenesis and play a role in cancer metastasis [15]. However, the effects of ERK1/2 signaling in IL-1β-induced inflammation-associated metastasis, and the synergistic effect of both growth and inflammatory factors on metastasis in IBDC, have not been well studied.

In this study, the roles of ERK1/2 in IBDC metastasis, and the function of EGF and IL-1β-induced ERK1/2-mediated signaling, were investigated in IBDC cells. We observed that activated phosphorylated ERK1/2 was associated with a higher TNM stage and the presence of lymph node metastasis in IBDC. Additionally, in vitro studies indicated that the activation of ERK-1/2 may increase the metastatic ability of IBDC cells, and *in vivo* investigations in IBDC tissue samples showed that the expression of p-ERK1/2 had good levels of correlation with the levels of EGF in addition to IL-1β, matrix metalloproteinase (MMP-9) and c-fos (AP-1). Growth and inflammatory factors synergistically induced IBDC cell migration and invasion via activation of the ERK1/2 signaling pathway, leading to the activation of AP-1 and increased matrix MMP-9 expression and activity.

## Materials and methods

### Tissue samples

The paraffin embedded blocks for 80 cases of invasive breast ductal carcinomas (IBDC) were obtained from Fuzhou General Hospital (Fuzhou, Fujian). The tissue samples were used with the consent of all patients. This study was approved by the Ethics Committee of Fuzhou General Hospital.

### Immunohistochemistry for phosphorylation of ERK1/2, EGF, IL-1β, EGF plus IL-1β, MMP-9 and c-fos

To assess the level of ERK1/2 phosphorylation (p-ERK1/2) by using immunohistochemical detection in the 80 cases of IBDC, we used previously described methods [16], with the use of a specific anti-p-ERK1/2 antibody (1:100 dilution, Cell Signaling Company, Danvers, MA, USA). The staining results were assessed on a four-tier scale based on that described by Ju and Ebert [17,18]: negative, no staining; 1+, weak staining; 2+, moderate staining; 3+, strong staining. Staining intensities ≥1 were considered positive. Statistical significance was evaluated by the Wilcoxon signed-rank test, Chi-square test and the partition of Chi-square test. To assess the level of EGF, IL-1β, EGF plus IL-1β, MMP-9 and c-fos in IBDC tissues by immunohistochemistry (IHC), we used the same method described above. Anti-MMP-9 and c-fos antibodies used for IHC were from Abcam (Cambridge, MA, USA); Anti-human IL-1β and EGF antibodies were from Santa Cruz (Santa Cruz, CA, USA) and Biosynthesis Biotechnology Co. (Beijing, China). Spearman’s method was used to analyze the correlation in expression levels of p-ERK1/2 with EGF plus IL-1β, MMP-9 or c-fos in IBDC tissue samples.

### Cell culture and transfection with siRNA

BT474 cells (American Type Culture Collection, Manassas, VA) were grown in RPMI 1640 medium (Invitrogen, Carlsbad, CA, USA) containing 10% fetal bovine serum (FBS) at 37°C in an incubator containing 5% CO_2_. SiRNA against ERK1/2 (Cell Signaling) or control siRNA (scrambled sequence siRNA was used as nonsilencing control siRNA) (Cell Signaling) was transfected into cells with Lipofectamine 2000 according to the manufacturer’s instructions.

### Western blotting for ERK1/2 and MMP-9

Western blotting for the expression of ERK1/2, p-ERK1/2 and MMP-9 in BT474 cells was conducted using previously described methods [19,20]. Briefly, 12% SDS-PAGE was used to detect the proteins. After the proteins were transferred onto PVDF membranes (Amersham Bioscience, Piscataway, NJ, USA) and incubated with the rabbit anti-human ERK1/2 or p-ERK1/2 (1:1,000 dilution, Cell Signaling) or mouse anti-human MMP-9 antibody (1:1,000 dilution, Abcam). The primary antibody was detected by a horseradish peroxidase-conjugated goat anti-rabbit or mouse secondary antibody (1:2,000 dilution, Santa Cruz). The immunoreactive protein bands were visualized with enhanced chemiluminescent (ECL, Amersham). Anti-β-actin (1:6,000 dilution, Sigma Company) was used as a control for the Western blots.

### Cell migration and invasion assay

For the invasion assay of BT474 cells, we used methods described by Sumida et al. [21]. Millicell Hanging Cell Invasion Chambers with 8-μm pore filter (Millipore Corporation) were coated with 12 μL of ice-cold Matrigel (7.5 mg/mL protein; Becton Dickinson Labware, Bedford, MA). BT474 cells (50,000 per well) were added to the upper chamber of these matrigel chambers in 200 μl serum-free RPMI 1640 medium with 20 ng/ml human EGF, 20 ng/ml IL-1β (R&D Systems), and both or neither. Cells were then placed into 24-well plates in RPMI 1640 medium containing 10% FBS. To evaluate the role of the U0126 inhibitor, cells were pre-treated with the reagent for 3 h, and the stimulations were then performed. To evaluate the role of ERK1/2 siRNA in cell migration and invasion, BT474 cells were transfected with scrambled siRNA or ERK1/2 siRNA for 36 h. Following this, the transfected cells were seeded at a density of 50,000 per well and then in 200 μl of serum-free medium for the stimulation. When the 22-h incubation was completed, cells were fixed with methanol and stained with Giemsa. Cotton tips were used to remove the cells that remained in the matrigel or attached to the upper side of the filter. Light microscopy was used to count the cells on the lower side of the filter. The assays were performed in duplicate, and the results were then averaged.

The methods used for the migration assay were almost the same as for the invasion assay described above, except no matrigel was used to coat the well and the incubation time was 16 h.

### RT-PCR assay

Total RNA was extracted from BT474 cells with the Trizol reagent (Invitrogen). The expression levels of MMP-9 mRNA were detected by first reverse-transcribing the total RNA, followed by PCR with the following primers: forward, 5^′^- CAGTCCACCCTTGTGCTCTTC-3^′^, reverse, 5^′^- TGCCACCCGAGTGTAACCAT -3^′^ for MMP-9. The expression levels of GAPDH mRNA in each sample were used as controls, and primers used for amplification of GAPDH mRNA were as follows: forward, 5^′^-GAGTCAACGGATTTGGTCGT-3^′^, reverse, 5^′^-TTGATTTTGGAGGGATCTCG-3^′^.

### MMP-9 zymography assay

MMP-9 protease activities in the concentrated supernatant medium of BT474 cells were detected by zymography. Briefly, 8% SDS-PAGE containing gelatin zymogram gels (Applygen Technologies Inc, Beijing, China.) were used to separate the proteins with electrophoresis. Renaturing and developing the gels were performed according to the manufacturer’s instructions, and the gels were then stained with Coomassie blue.

### AP-1 luciferase reporter gene assay

BT474 cells were transfected with AP-1 luc vector (1 μg) or AP-1 luc vector plus scrambled siRNA (50–200 nM) or ERK1/2 siRNA (50–200 nM) with Lipofectamine 2000. B-gal plasmid (containing–galactosidase reporter gene) was co-transfected with AP-1 reporter plasmids to serve as the control for transfection efficiency. Thirty-six hours after transfection, the cells were left untreated or were treated with 20 ng/ml of EGF, IL-1β or both for 12 h. The luciferase assay (for AP-1) and enzyme assay (for B-gal) were then performed according to the instructions of the Promega kit (Madison, WI, USA).

### Statistical analysis

Statistical significance of IHC for p-ERK1/2 was evaluated by the Wilcoxon signed-ranks test, Chi-square test, and the partition of Chi-square test. Spearman’s method was used to analyze the correlation in expression levels of p-ERK1/2 with EGF plus IL-1β, MMP-9 or c-fos in IBDC tissue samples. For other experiments, values are expressed as means ± SD, and independent-sample t-tests were performed to determine differences among groups. P-values <0.05 were considered statistically significant.

## Results

### Expression of phosphorylated ERK1/2 in invasive breast ductal carcinoma

**Figure 1:**
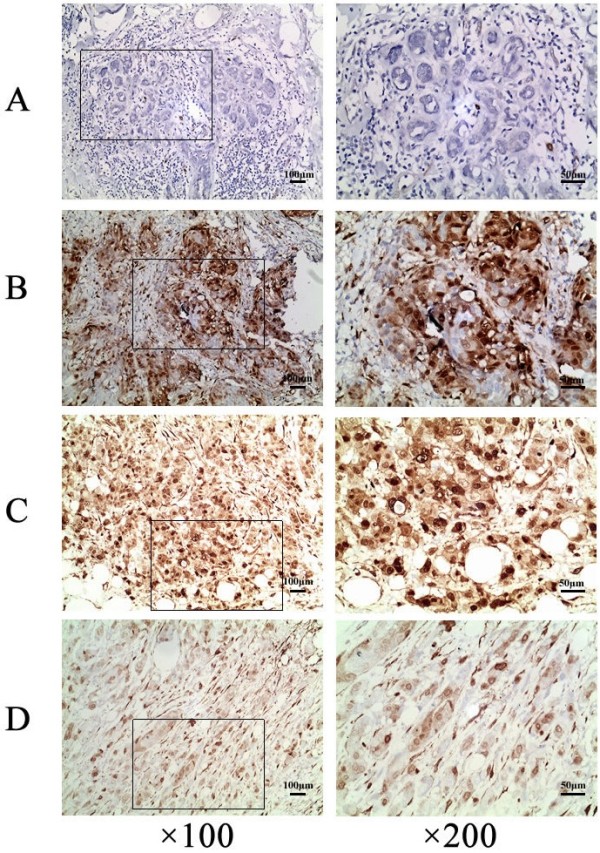
**Detection of p-ERK1/2 expression in IBDC and non-neoplastic tissues using immunohistochemistry.** P-ERK1/2 was not detected or very weakly expressed in non-neoplastic breast ductal cells (**A**), but was frequently expressed in both the cytoplasm and nucleus of IBDC cells (**B** to **D**). B to D represented different intensities of p-ERK1/2 positive staining in IBDC. Images in the left panel were ×100 magnification; and expanded on the right at ×200.

Activated ERK1/2 (p-ERK1/2) has been shown to be expressed in many different human cancers [22], and is likely to play a role in cancer cell growth and metastasis. To investigate whether p-ERK was expressed in IBDC, and to analyze the relationship between the expression of p-ERK and the clinicopathological features of IBDC, paraffin-embedded tissues from 80 cases of IBDC were examined by IHC using an antibody specific for p-ERK1/2. Positive p-ERK1/2 staining was detected in both the cytoplasm and nucleus of IBDC cells (Figure 1). p-ERK1/2 was positively expressed in 58/80 cases of IBDC (72.5%, Table 1) compared to 11/80 (13.75%) of the non-neoplastic tissues (*P* < 0.05, Wilcoxon signed-rank test). The expression of p-ERK1/2 was not correlated with patient age or tumor size; however, the expression of p-ERK1/2 was closely associated with higher TNM stage and lymph node metastasis in IBDC (Table 1).

**Table 1:** Immunohistochemical detection of phospho-ERK1/2 expression in invasive breast ductal cancer

**Factor**	**N**	**p-ERK1/2 positive n (%)**	**p-ERK1/2 negative n (%)**	***P *value**
Total	80	58 (72.5)	22 (27.5)	<0.05
Age				>0.05
<50	47	36 (76.6)	11 (23.4)	
≥50	33	22 (66.7)	11 (33.3)	
TNM staged				<0.05
T1	28	16 (57.14)	12 (42.86)	T1:T2+T3, P<0.05
T2	43	34 (79.07)	9 (20.93)	
T3	9	8 (88.89)	1 (11.11)	
Tumor size*				>0.05
≥3 cm	33	23 (69.7)	10 (30.3)	
<3 cm	47	35 (74.5)	12 (25.5)	
Lymph node				<0.01
Positive^△^	44	40 (90.9)	4 (9.1)	
Negative	36	18 (50)	18 (50)	

^*^ Tumor size was expressed as length × width; tumors with length or width ≥3 cm were classified as >3 cm; tumors with both length and width <3 cm were classified as <3 cm.

^Δ^ Patient with at least one positive lymph node.

There was no significant difference in the expression of p-ERK1/2 in tumors ≥3 cm (23/33, 69.7%), those sized <3 cm (35/47, 74.5%; *P* > 0.05, Chi-square test), or the tumors of patients aged ≥50 yr (22/33, 66.7%) or <50 yr (36/47, 76.6%; *P* > 0.05, Chi-square test). The expression of p-ERK1/2 varied significantly with TNM stage (*P* < 0.05, Chi-square test). p-ERK1/2 expression was detected in all three TNM stages, and was more frequent in stage T2 and T3 tumors than T1 tumors (*P* < 0.05, partition of Chi-square test). p-ERK expression was significantly different in the tumors of patients with and without lymph node metastasis (*P* < 0.01, Chi-square test), which indicated that the expression of p-ERK1/2 correlates with metastasis and poorer prognosis in IBDC.

### EGF-induced ERK1/2 activation and IBDC cell migration and invasion can be attenuated by ERK1/2 siRNA or U0126

**Figure 2:**
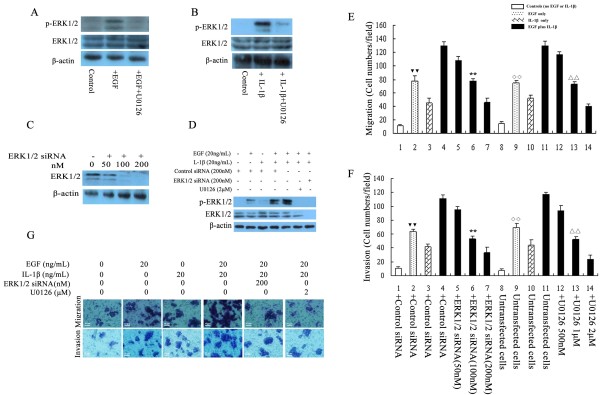
**EGF and IL-1β synergistically promote ERK1/2-mediated IBDC cell migration and invasion.A**: Increased expression of activated ERK1/2 was detected in BT474 cells after stimulation with EGF. Western blotting demonstrating that pre-treatment with U0126 significantly inhibited EGF-induced ERK1/2 activation in BT474 cells. **B**, Increased expression of activated ERK1/2 was detected after the stimulation of BT474 cells with IL-1β. Western blotting demonstrating that pre-treatment with U0126 significantly inhibited IL-1β-induced ERK1/2 activation in BT474 cells. **C**, Western blots confirmed that the transfection of ERK1/2 siRNA dose-dependently reduced ERK1/2 expression in BT474 cells. **D**, Stronger activation of ERK1/2 was detected in EGF plus IL-1β-stimulated BT474 cells (an approximate 2- to 3-fold increase) compared to EGF or IL-1β alone. ERK1/2 siRNA and U0126 inhibited both EGF and IL-1β-induced ERK1/2 activation. **E** and **F**, Increased numbers of migrating and invading BT474 cells were observed after stimulation with EGF plus IL-1β, leading to a 2-fold increase compared to either EGF or IL-1β alone. ERK1/2 siRNA and U0126 inhibited EGF plus IL-1β-induced cell migration and invasion. **G**, Representative light microscopy images of BT474 cell migration and invasion in the Transwell assay. ^**^ and ^▼▼^, *P* < 0.05 vs. siRNA negative control-transfected cells stimulated with EGF plus IL-1β; ^ΔΔ^ and ^**◇◇**^, *P* < 0.05 vs. untransfected cells stimulated with EGF plus IL-1β. Bars are the mean ± SD of migrating and invading cells. Cells were counted as the number of cells per field of view.

Growth factors can activate ERK1/2, and ERK1/2 activation is closely related to cancer cell migration and invasion, which may be regulated by the upstream kinase MEK1/2 via the growth factor-induced metastasis-associated pathway [23]. Therefore, we investigated the function of the representative growth factor, EGF and ERK1/2 signaling, in IBDC cells. BT474 cells were stimulated with EGF for 30 min. As expected, EGF-induced ERK1/2 activation was demonstrated by the detection of p-ERK1/2 in the cells (Figure 2A).

To determine whether EGF affects the migration and invasion of IBDC cells via ERK1/2 signaling, BT474 cells were treated with EGF in the presence or absence of ERK1/2 siRNA or the MEK/ERK pathway inhibitor, U0126. The Transwell migration assay revealed that EGF increased the migration and invasion of the cells; however, EGF-induced BT474 cell migration and invasion were significantly attenuated in a dose-dependent manner by the knockdown of ERK1/2 using 50–200 nM ERK1/2 siRNA (Figures 2C, E and F). Similarly, U0126 significantly decreased EGF-induced cell migration and invasion in a dose-dependent manner (Figures 2E and F). These data strongly indicated that ERK1/2 plays an important role in growth factor-induced IBDC cell migration and invasion, and demonstrated that ERK1/2-mediated IBDC cell migration and invasion are regulated by MEK1/2.

### IL-1ß-induced ERK1/2 activation and IBDC cell migration and invasion can be attenuated by ERK1/2 siRNA or U0126

It is established that inflammatory microenvironment signaling plays an important role in cancer progression, including tumor metastasis [24]. ERK1/2 are essential molecules associated with cancer metastasis. Many previous studies have focused on the role of ERK1/2 in growth factor-induced metastasis; however, ERK1/2 can also be partially activated by pro-inflammatory factors [13]. The contribution of ERK1/2 to inflammatory signal pathway-mediated metastasis has not been well studied. In order to understand the role of ERK1/2 in inflammatory factor-induced IBDC cell metastasis, BT474 cells were treated with the major cytokine IL-1β. IL-1β has been reported to activate ERK1/2 in several cell types, including cancer cells [25,26]. As expected, IL-1β activated ERK1/2, as p-ERK1/2 could be detected in BT474 cells 30 min after IL-1β stimulation (Figure 2B).

To examine the contribution of IL-1β to IBDC cell migration and invasion, BT474 cells were treated with or without IL-1β. Increased cell migration and invasion were observed in cells treated with IL-1β. Transwell assays demonstrated that knockdown of ERK1/2 expression using siRNAs attenuated IL-1β-induced cell migration and invasion in a dose-dependent manner (Figures 2E and F). The MEK/ERK inhibitor U0126 also significantly inhibited IL-1β-induced BT474 cell migration and invasion (Figures 2E and F), indicating that IL-1β-induced IBDC cell metastasis are dependent on the MEK/ERK signaling pathway, and also that ERK1/2 contributes to inflammatory factor-associated IBDC cell migration and invasion.

### EGF and IL-1β synergistically promote ERK1/2-mediated-IBDC cell migration and invasion

Our results provided strong evidence to suggest that both growth factor and inflammatory factor stimulation could increase IBDC cell migration and invasion; however, it was not clear whether growth and inflammatory factors could exert a synergistic effect. Therefore, BT474 cells were stimulated with 20 ng/mL EGF plus 20 ng/mL IL-1β. As shown in Figure 2D, a two to three-fold increase in p-ERK1/2 expression was detected when cells were co-stimulated with both EGF and IL-1β, compared to either EGF, or IL-1β alone. Activation of ERK1/2 by EGF or IL-1β was almost completely blocked by ERK1/2 siRNA or the MEK/ERK pathway inhibitor U0126. Co-stimulation with EGF and IL-1β significantly increased the migration and invasion of BT474 cells by a factor of about two-fold, compared to cells treated with either EGF or IL-1β alone (Figures 2E and F). Representative light microscope images of BT474 cell migration and invasion in the Transwell assay were displayed in Figure 2G.

### EGF and IL-1β synergistically upregulate MMP-9 in IBDC cells via the ERK1/2 pathway

**Figure 3:**
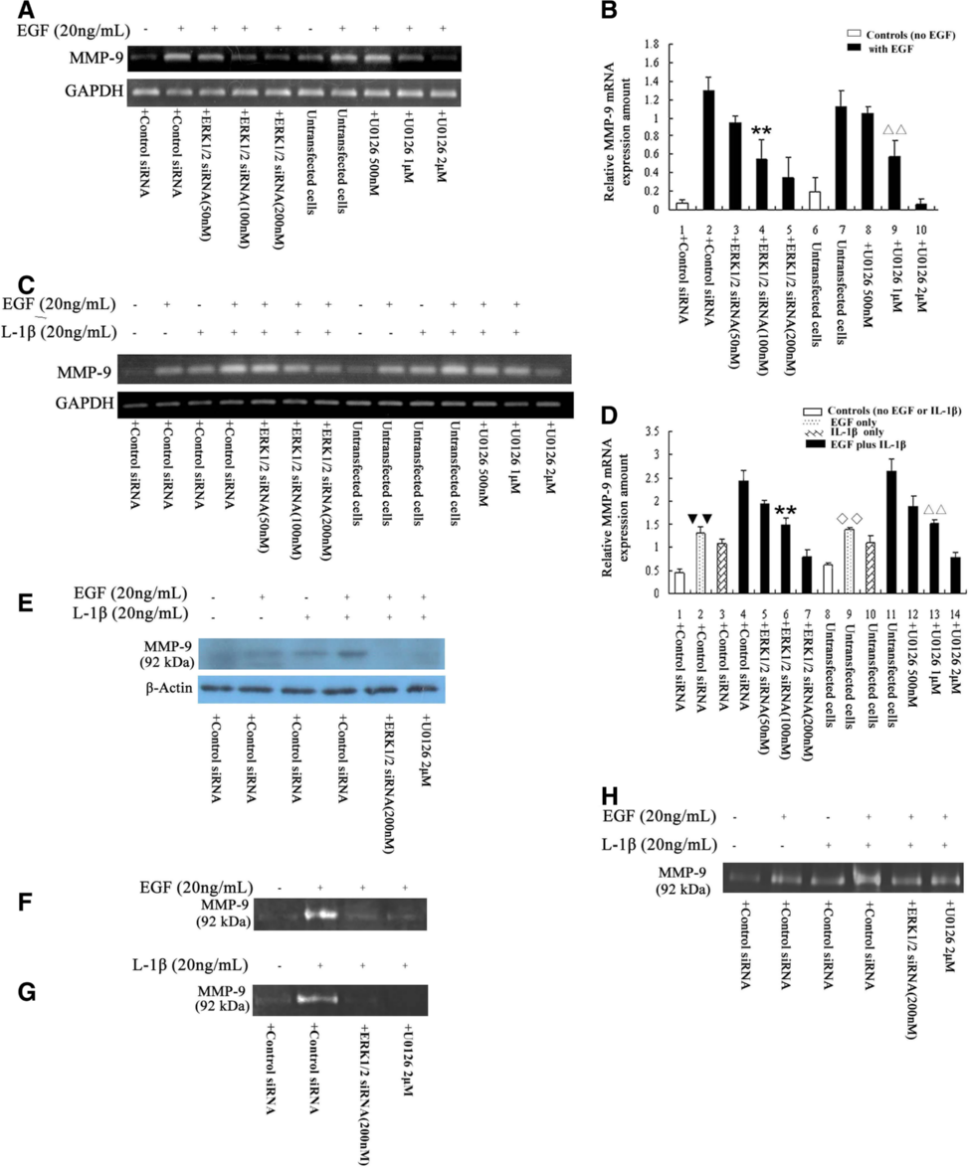
**EGF plus IL-1β synergistically upregulate MMP-9 in IBDC cells via the ERK1/2 pathway.A**, RT-PCR showing that *MMP-9* mRNA was expressed in BT474 cells and increased after EGF treatment. Knockdown of ERK1/2 using siRNA or pre-treatment with U0126 dose-dependently attenuated EGF-induced *MMP-9* mRNA upregulation in the cells. **B**, Bars indicate the relative expression levels of *MMP-9* mRNA normalized to *GAPDH* mRNA. ** *P* < 0.05 vs. control siRNA-transfected cells stimulated with EGF; ^ΔΔ^*P* < 0.05 vs. untransfected cells stimulated with EGF. **C**, IL-1β also increased *MMP-9* mRNA expression in BT474 cells; EGF plus IL-1β synergistically increased MMP-9 expression compared to either EGF or IL-1β stimulation alone; this effect could be inhibited by ERK1/2 siRNA or pre-treatment with U0126. **D**, Bars indicate the relative expression levels of *MMP-9* mRNA normalized to *GAPDH* mRNA. ** and ^▼▼^, *P* < 0.05 vs. siRNA negative control-transfected cells stimulated with EGF plus IL-1β; ^ΔΔ^ and ^**◇◇**^, *P* < 0.05 vs. untransfected cells stimulated with EGF plus IL-1β. **E**, EGF plus IL-1β synergistically increased MMP-9 expression compared to either EGF or IL-1β stimulation alone as analyzed by Western blotting. **F** to **H**, MMP-9 activity was analyzed using zymography. **F**, EGF increased MMP-9 activity; this effect could be attenuated by both ERK1/2 siRNA and U0126 in the cells. **G**, IL-1β also increased *MMP-9* activity in BT474 cells; **H**, EGF plus IL-1β synergistically increased MMP-9 activity compared to either EGF or IL-1β stimulation alone.

Many studies have demonstrated that the upregulation of MMP-9 is associated with increased cancer cell migration and invasion [27]. To test whether MMP-9 contributes to ERK1/2-mediated IBDC cell metastasis in response to EGF or IL-1β, RT-PCR, Western blotting and zymography assays were performed to detect the expression and activity of MMP-9 in BT474 cells. As expected, EGF increased MMP-9 expression and enzymatic activity in the cells, as demonstrated by the increased expression of *MMP-9* mRNA, protein and disappearance of the MMP-9 substrate bands in the zymography assay. The knockdown of ERK1/2 by siRNA or the inhibition of ERK1/2 activation by U0126 significantly reduced EGF-induced *MMP-9* mRNA and protein expression in a dose-dependent manner (*P* < 0.05 compared with control siRNA or untransfected cells, independent sample *t-*test), and attenuated the EGF-induced increase in MMP-9 activity (Figures 3A to F, and H, respectively). IL-1β alone also induced the upregulation of MMP-9 in BT474 cells; however, EGF in the presence of IL-1β synergistically increased both MMP-9 expression and activity by approximately 2-fold compared to EGF or IL-1β alone (Figures 3C to E, and G to H).

### Knockdown of ERK1/2 inhibits the synergistic activation of activator protein-1 in IBDC cells by EGF and IL-1β

**Figure 4:**
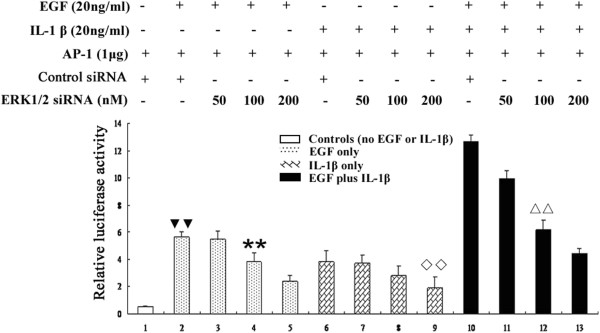
**Knockdown of ERK1/2 inhibits the synergistic activation of AP-1 by EGF and IL-1β in IBDC cells.** EGF and IL-1β stimulation increased the activity of an AP-1 luciferase reporter gene in BT474 cells; however, EGF plus IL-1β synergistically increased AP-1 luciferase activity. Transfection of BT474 cells with ERK1/2 siRNA decreased EGF or IL-1β, or EGF plus IL-1β-induced AP-1 activation in a dose-dependent manner. ** or ^**◇◇**^, *P* < 0.05 vs. control siRNA and AP-1 luc-transfected cells stimulated with EGF or IL-1β alone; ^▼▼^ and ^ΔΔ^, *P* < 0.05 vs. control siRNA and AP-1 luc-transfected cells stimulated with EGF plus IL-1β; relative luciferase activity was normalized against B-gal.

The transcription factor activator protein-1 (AP-1) is one of the most important regulators of *MMP-9* expression [28]. Our data showed that both EGF and IL-1β upregulated MMP-9, and ERK1/2 has been demonstrated to play critical role in the regulation of AP-1 activation. In order to investigate whether the synergistic mechanism by which EGF and IL-1β upregulated MMP-9 via ERK1/2 was dependent on AP-1, AP-1 activation was detected using an AP-1 luciferase reporter gene assay. As shown in Figure 4, EGF treatment increased AP-1 luciferase activity in BT474 cells, and IL-1β also induced the activation of AP-1 in BT474 cells. The knockdown of ERK1/2 by siRNA significantly reduced both EGF- and IL-1β-induced AP-1 activation in a dose-dependent manner (*P* < 0.05 compared with control siRNA, independent sample *t-*test). Co-stimulation with EGF and IL-1β synergistically increased AP-1 activity by a factor of approximately 2-fold, compared to either EGF or IL-1β stimulation alone (Figure 4), and the inhibition of ERK1/2 using siRNA reduced AP-1 reporter gene activity in cells treated with EGF plus IL-1β. A dose-dependent decrease in AP-1 luciferase activity was detected in BT474 cells transfected with different amounts of ERK1/2 siRNA (50–200 nM) and the AP-1 luciferase reporter gene plasmid (Figure 4).

### Relationship between of the expression level of p-ERK1/2, EGF plus IL-1β, MMP-9 and AP-1 in IBDC tissue samples

**Figure 5:**
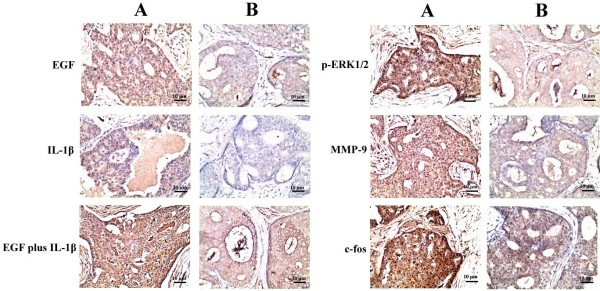
**The expression of p-ERK1/2 was closely related to EGF with IL-1β, MMP-9 and AP-1 in IBDC tissue samples.A**, Strong expression levels of EGF, IL-1β, and EGF plus IL-1β, stronger expression of p-ERK1/2, MMP-9 and c-fos in IBDC tissue sample. **B**, Weaker expression levels of EGF, IL-1β, and EGF plus IL-1β, weaker expression levels of p-ERK1/2, MMP-9 and c-fos in IBDC tissue sample.

In order to understand the relationship between the expression of p-ERK1/2 with proteins of interest in IBDC tissue samples, IHC was used to assay the expression of p-ERK1/2, EGF, IL-1β (or IL-1β plus EGF), MMP-9 and AP-1(c-fos). The representative IHC results from two cases of IBDC were displayed in Figure 5. As shown in Figure 5, though the expression of p-ERK1/2 correlated with the levels of EGF alone (r=0.638, *p*<0.01) or IL-β alone (r=0.564, *p*<0.05), the expression of p-ERK1/2 correlated well with levels of IL-1β plus EGF, in addition to MMP-9 and c-fos. There was a significant correlation between increasing p-ERK1/2 expression levels and the elevated expression of EGF plus IL-1β, MMP-9 or c-fos in IBDC tissue samples (r = 0.83, *p*<0.001; r=0.86, *p*<0.001 and r=0.77, *p*<0.001, respectively). The data demonstrated that higher levels of EGF with IL-1β positively correlated with increased levels of p-ERK1/2, MMP-9 and c-fos expression in IBDC *in vivo*.

## Discussion

In the present work, we demonstrate for the first time that p-ERK1/2 may be involved in the metastasis of IBDC. Additionally, growth and inflammatory factors may synergistically induce IBDC metastasis by increasing cell migration and invasion via the activation of ERK1/2 signaling, due to the AP-1-dependent upregulation of MMP-9.

ERK1/2 are important regulators of progression and metastasis in a variety of cancers via the MEK/ERK/AP-1 signaling pathway [23,28]. However, it remains unknown as to whether ERK1/2 plays a role in IBDC metastasis. In this study, we detected the expression of activated ERK1/2 in the majority of IBDC tissue samples using IHC assays, and found that the expression of p-ERK1/2 was closely related with a higher TNM stage and the presence of lymph node metastasis. Therefore, activated ERK1/2 may correlate with a poorer prognosis in IBDC. Karroum *et al.* previously reported that the expression of activated ERK1/2 was associated with cell migration and the formation of a tubular network of resistant MCF-7 breast cancer cells via a mechanism linked to the activation of MMP-9 [29].

The involvement of growth factors in cancer growth and metastasis has been widely documented. However, little is known about the role of inflammatory signaling pathways in metastasis, or the combined action of growth factors and inflammatory factors in IBDC cells. To gain an insight into the function of growth factors and inflammatory factors in IBDC metastasis, the representative growth factor, EGF, which can activate ERK1/2, and one of the most common inflammatory factors, IL-1β, were investigated. Consistent with previous results in other cancer cell lines, EGF increased IBDC cell migration and invasion via a mechanism regulated by ERK1/2. Importantly, we also demonstrated for the first time that IL-1β also enhanced IBDC cell migration and invasion, and the presence of EGF and IL-1β synergistically increased IBDC cell migration and invasion via the ERK1/2 pathway. Therefore, ERK1/2 signaling plays an important role in inflammatory factor-associated IBDC cell migration and invasion.

ERK1/2 is activated by MEK1/2 [30], and we confirmed that the inhibition of ERK1/2 signaling using the MEK1/2 inhibitor, U0126, or ERK1/2 siRNA significantly attenuated EGF-induced cancer cell migration and invasion in a dose-dependent manner. The breakdown of the extracellular matrix (ECM) is a crucial step during the metastatic process [31], and many studies have shown that MMPs play an important role in ECM degradation [30,31]. It is well known that ERK1/2 mediates cancer cell metastasis by regulating MMP-9 expression and activity. MMP-9 is one of the most important MMPs and is closely related to cancer invasion and metastasis as a result of its strong ECM proteolytic activity [27,28]. MMP-9 induces cell migration and invasion by degrading collagen in the basement membrane, which allows cancer cells to detach from the ECM and invade surrounding tissues. In this study, we demonstrated that EGF, in combination with IL-1β, induced IBDC cell migration and invasion via the activation of ERK1/2, which increased the expression and activation of MMP-9. The role of ERK1/2 in this process was confirmed by the fact that both siRNAs against ERK1/2 and the ERK/MEK inhibitor attenuated EGF and IL-1β-induced cell migration and invasion.

ERK1/2 is well characterized as a serine/threonine protein kinase that regulates the activation of the transcription factor, AP-1 [32]. The activation of reporter genes, as indicated by increased luciferase activity, is one characteristic of gene activation *in vitro*. Indeed, ERK1/2 siRNA suppressed the ability of EGF and IL-1β to induce activation of an AP-1 reporter gene, which demonstrated that the EGF and IL-1β-induced activation of ERK1/2 led to the upregulation of MMP-9 by increasing the transcriptional activity of AP-1. More importantly, data from tissue samples of IBDC also confirmed that the expression of p-ERK1/2 correlated strongly with the levels of EGF plus IL-1β, MMP-9, and c-fos (AP-1) *in vivo*.

## Conclusions

This study demonstrates that the expression of p-ERK1/2 in IBDC is closely related to lymph node metastasis and high tumor grade, which are indicative of poor patient prognosis. We also showed that ERK1/2 may functionally regulate metastasis, as growth and inflammatory factors synergistically increased IBDC cell migration and invasion via the ERK1/2 signaling pathway. This led to the activation of AP-1 and the upregulation of MMP-9. This study suggests that ERK1/2 may represent a useful therapeutic target for IBDC.

## Abbreviations

ERK: Extracellular signal-regulated kinase; IBDC: Invasive breast ductal carcinomas; MAPK: Mitogen-activated protein kinase; MEK: Mitogen-activated protein kinase kinase; MMP-9: Matrix metalloproteinase-9; AP-1: Activator protein 1; EGF: Epidermal growth factor; IL-1β: Interleukin -1β; p-ERK1/2: Phosphorylation ERK1/2; ECM: Extracellular matrix.

## Competing interests

The authors declare that they have no competing interests.

## Authors’ contributions

LM performed the experiments and data analysis. FL participated in guiding some experiments. ZZ, FX and WL carried out immunohistochemical samples collecting and the results analysis. LW provided tissue samples and clinical data. JH performed some of the experimental studies. FZ participated in some experiment design and data analysis. YX performed some of the experiments. QH performed some experiments, and contributed to design the studies, interpret the data and write the manuscript. All authors read and approved the final manuscript.
